# Sedation protocols *versus* daily sedation interruption: a systematic review and meta-analysis

**DOI:** 10.5935/0103-507X.20160078

**Published:** 2016

**Authors:** Antonio Paulo Nassar Junior, Marcelo Park

**Affiliations:** 1 Discipline of Clinical Emergency, Hospital das Clínicas, Faculdade de Medicina, Universidade de São Paulo - São Paulo (SP), Brazil.

**Keywords:** Conscious sedation, Clinical protocols, Respiration, artificial, Critical care, Sedação consciente, Protocolos clínicos, Respiração artificial, Cuidados críticos

## Abstract

**Objective:**

The aim of this study was to systematically review studies that compared a mild target sedation protocol with daily sedation interruption and to perform a meta-analysis with the data presented in these studies.

**Methods:**

We searched Medline, Scopus and Web of Science databases to identify randomized clinical trials comparing sedation protocols with daily sedation interruption in critically ill patients requiring mechanical ventilation. The primary outcome was mortality in the intensive care unit.

**Results:**

Seven studies were included, with a total of 892 patients. Mortality in the intensive care unit did not differ between the sedation protocol and daily sedation interruption groups (odds ratio [OR] = 0.81; 95% confidence interval [CI] 0.60 - 1.10; I^2^ = 0%). Hospital mortality, duration of mechanical ventilation, intensive care unit and hospital length of stay did not differ between the groups either. Sedation protocols were associated with an increase in the number of days free of mechanical ventilation (mean difference = 6.70 days; 95%CI 1.09 - 12.31 days; I^2^ = 87.2%) and a shorter duration of hospital length of stay (mean difference = -5.05 days, 95%CI -9.98 - -0.11 days; I^2^ = 69%). There were no differences in regard to accidental extubation, extubation failure and the occurrence of delirium.

**Conclusion:**

Sedation protocols and daily sedation interruption do not appear to differ in regard to the majority of analyzed outcomes. The only differences found were small and had a high degree of heterogeneity.

## Abstract (pt)

**Objetivo:**

Revisar, de forma sistemática, os estudos que compararam um protocolo com alvo de sedação leve e a interrupção diária da sedação, bem como realizar uma metanálise com os dados apresentados nestes estudos.

**Métodos:**

Foram realizadas buscas nas bases de dados *Medline, Scopus* e *Web of Science* para a identificação estudos clínicos randomizados que compararam protocolos de sedação com a interrupção diária da sedação em pacientes críticos com necessidade de ventilação mecânica. O desfecho primário analisado foi mortalidade na unidade de terapia intensiva.

**Resultados:**

Foram incluídos 7 estudos, com total de 892 pacientes. A mortalidade na unidade de terapia intensiva não foi diferente entre os grupos protocolo de sedação ou interrupção diária da sedação (OR = 0,81; IC 95% 0,60 - 1,10; I^2^ = 0%). Mortalidade hospitalar, duração da ventilação mecânica e da internação na unidade de terapia intensiva também não foram diferentes dentre os grupos. Os protocolos de sedação associaram-se a um aumento do número de dias livres de ventilação mecânica (diferença média = 6,70 dias; IC95% 1,09 - 12,31 dias; I^2^ = 87,2%). Os protocolos de sedação associaram-se a uma menor duração da internação hospitalar (diferença média = -5,05 dias; IC 95% -9,98 - -0,11 dias; I^2^ = 69%). Não houve diferenças quanto à extubação acidental, à falha de extubação e à ocorrência de *delirium*.

**Conclusão:**

Protocolos de sedação e interrupção diária da sedação não parecem diferentes quanto à maioria dos desfechos analisados. As diferenças encontradas foram pequenas e com um elevado grau de heterogeneidade.

## INTRODUCTION

Proper sedation is an important component in the care of critically ill patients requiring mechanical ventilation. Deep sedation levels are associated with several negative outcomes, such as increased time on mechanical ventilation,^(1)^ delirium,^(2)^ memory disturbances,^(3)^ and higher mortality in the short^(4)^ and long term.^(5)^

The deleterious effects of deep sedation can be minimized by employing a strategy of sedation protocols that target lighter sedation levels^(6,7)^ and the daily interruption of sedative infusion.^(8,9)^ The effects of these strategies have been assessed in two systematic reviews in which the included study control groups consisted of patients who received "usual" care in relation to the sedation of patients on mechanical ventilation. The first systematic review that included observational and randomized studies showed that most of the studies suggested a reduction in the duration of mechanical ventilation and ICU and hospital length of stay.^(10)^ The second systematic review included only randomized studies and pooled their results into a meta-analysis, which indicated that there were reduced ICU and hospital length of stay and reduced mortality with the use of both sedation reduction strategies.^(11)^ Another meta-analysis also suggested that the two sedation minimization strategies were not associated with higher incidences of post-traumatic stress in the long term,^(12)^ which was a fear that had been raised when the first study on daily sedation interruption was published.^(13)^

Therefore, protocols targeting either a light sedation level or daily sedative infusion interruption should be adopted to reduce the deleterious effects of excessive sedation.^(14)^ However, the use of these strategies is still far from universal,^(15)^ and it is unclear whether one of the two is more effective than the other.

The objective of this study was to systematically review studies that compared a light target sedation protocol with daily sedation interruption.

## METHODS

### Search strategy

Searches of the Medline (via PubMed), Scopus and Web of Science databases were performed. The studies were obtained by combining the following keywords: ("sedation" OR "sedatives") AND ("critical care" OR "intensive care" OR "critically ill") AND ("daily interruption"). Additional studies were sought based on the references of included studies and personal files. There was no language restriction. The searches were limited to randomized clinical studies performed on adults and published up to February 4, 2016. Titles and abstracts were assessed for eligibility. The full texts of potentially relevant articles were analyzed. The eligibility assessment was conducted by the authors, and disagreements were resolved by consensus. Preferred Reporting Items for Systematic Review and Meta-Analysis (PRISMA) guidelines were used as a guide.^(16)^ The systematic review was recorded in the PROSPERO database (CRD 42014014121). As the study is a literature review, there was no need for Ethics Committee approval.

### Study selection

Studies that met the following criteria were included: those comparing a protocol with a predefined sedation scale target with daily sedative infusion interruption; and those assessing any of the following outcomes: mortality in intensive care, duration of mechanical ventilation, days free of mechanical ventilation in 28 days and ICU length of stay.

### Data extraction

The authors extracted the following data independently using a specific form: country where the study was conducted, year of publication, study design, number of patients included in each study group, description of the sedation protocol and the manner in which daily sedation interruption was conducted, ICU and hospital mortality, duration of mechanical ventilation, days free of mechanical ventilation in 28 days, ICU and hospital length of ICU stay, delirium, accidental extubation rates and extubation failure (reintubation within 48 hours). Authors of included studies were contacted by e-mail to obtain information about missing data from the publications.

### Evaluation of study quality

Study quality was assessed by the Cochrane risk of bias assessment tool for clinical studies. The risk of bias was assessed as "low", "uncertain" or "high" in the following areas: generation of random sequence, allocation concealment, blinding of participants and professionals, blinding of outcome assessors, incomplete outcomes, selective outcome reporting, and other sources of bias. Disagreements were resolved by consensus.

### Outcomes

The primary outcome was mortality in the ICU. Secondary outcomes were duration of mechanical ventilation, days free of mechanical ventilation in 28 days, hospital mortality, ICU and hospital length of stay, prevalence of delirium, and accidental extubation and extubation failure rates (reintubation within 48 hours after extubation).

### Statistical analysis

A random effects model was used due to the variability among studies regarding samples and how the interventions were applied. The differences between groups were expressed as odds ratios (OR) for categorical variables and as mean differences (MD) for continuous variables, both with 95% confidence intervals (95%CI). The reference group for the analysis was always "sedation protocol." Heterogeneity was assessed using the I^2^ statistic and was classified as low (< 25%), moderate (25 - 50%) or high (> 50%). The analyses were performed using *R* software version 3.3.1, with *R* Studio version 0.99.902, and the meta package (version 4.4.0) developed by Guido Schwazer (http://cran.rproject.org/web/packages/meta/meta.pdf).

## RESULTS

### Study characteristics

**Figure 1:**
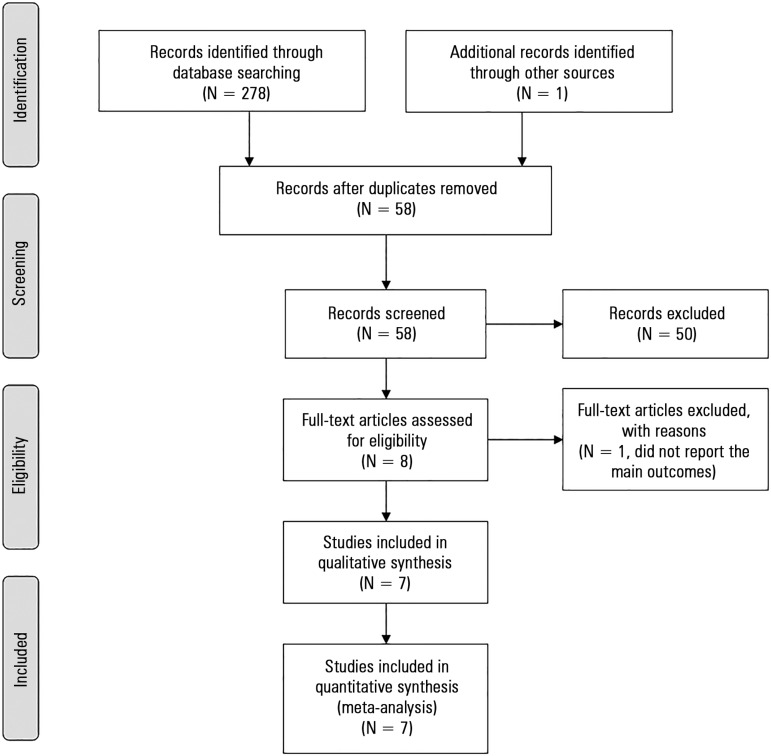
Study flowchart.

A total of 279 references were identified by the search strategies, eight full-text articles were assessed for eligibility. In total, seven studies^(17-23)^ were included; one was excluded, as it did not report any of the outcomes of interest^(24)^ (Figure 1).

The characteristics of the included studies are described in table 1. In general, the studies were small, and only one was a multicenter study. The goal was light to moderate sedation in all studies. The Sedation-Agitation Scale (SAS), the Ramsay scale and the Richmond Agitation Sedation Scale (RASS) were used in three, three and two studies, respectively. Only one of the studies had a deeper sedation level as its lower target (Ramsay 5).^(19)^ Descriptions of the sedation protocols and daily sedation interruption can be found in table 2.

**Table 1:** Study characteristics

Study	Country	Number of centers	Number of patients (protocol/daily interruption)	Sedation target
Mehta et al.^(17)^	Canada	1	33/32	SAS 3 - 4
de Wit et al.^(18)^	United States	1	38/36	RASS -2 - -3
Anifantaki et al.^(19)^	Greece	1	48/49	Ramsay 3 - 5
Strom et al.^(20)^	Denmark	1	70/70	Ramsay 3 - 4
Yiliaz et al.^(21)^	Turkey	1	25/25	Ramsay 3 - 4
Mehta et al.^(22)^	Canada and United States	16	209/214	SAS 3 - 4 or RASS -3 - 0
Nassar Junior e Park^(23)^	Brazil	1	30/30	SAS 3 - 4

SAS - Sedation Agitation Scale; RASS - Richmond Agitation Sedation Scale.

**Table 2:** Sedation protocol and daily interruption performed in each study

Study	Sedation protocol	Daily sedation interruption
Mehta et al.^(17)^	Midazolam and morphine (or fentanyl, if CrCl < 10mL/min) reduced every 15 - 30 minutes if SAS 1-2. Boluses were administered if there was agitation, and sedative and analgesic doses were increased. SAS was reassessed every 1 - 2 hours	The infusion of sedatives and opioids was maintained identically to the protocol, but sedatives and analgesics were turned off after 9 hours, and the patients were assessed for their ability to obey three out of four commands (open your eyes, follow the investigator with your eyes, shake hands and wiggle your toes). If the doctor felt that the patient needed to be sedated, sedation was reinitiated at half the dose. In this case, the protocol continued, targeting SAS 3-4. If it was decided that the patient would not receive any more sedatives, they were only resumed if the patient was at SAS 6 - 7
de Wit et al.^(18)^	Analgesia with morphine or fentanyl (if renal failure or hemodynamic instability) in bolus. If boluses were frequent, continuous infusion began. Sedation followed the same pattern, with the use of midazolam or lorazepam. Where there was a need for continuous infusion, lorazepam or propofol were used if there was renal or hepatic failure and lorazepam and midazolam if there was hemodynamic instability. The analgesics and sedatives of patients with RASS 1 or 2 points below the target were reduced by 25 - 50% every 4 hours. If the RASS was more than two points below the target, the drugs were discontinued	The sedatives and opioids were turned off 48 hours after the beginning of mechanical ventilation. Patients were considered awake if they could follow three of four commands (open your eyes, follow the researcher, put out your tongue and shake hands). The resumption of sedatives was at the discretion of the investigators. Sedatives were restarted at half the dose if the patient was awake, agitated or had a change in vital signs (RR > 35ipm; SaO_2_ < 90%; HR > 140bpm or change of 20% in either direction; SBP > 180mmHg or < 90mmHg). The team had to target RASS -2 to -3 and performed sedative infusion in the absence of the investigators
Anifantaki et al.^(19)^	Sedatives (midazolam or propofol) and opioids (remifentanil) were adjusted to maintain Ramsay 3-5. The adjustments were performed every 2 minutes until the target was reached.	Sedative infusion was turned off after patient recruitment, but the remifentanil infusion was maintained at a rate of 0.05 - 0.25mg/hour. If the patient was agitated, presented respiratory distress, hemodynamic instability or neurological deterioration (e.g., increased ICP), sedatives and analgesics were reinitiated at half the previous dose
Strom et al.^(20)^	Analgesia with morphine. If discomfort was experienced, the team searched for reversible causes. If delirium was suspected, haloperidol was administered. If agitation was still present, propofol was initiated for 6 hours. After this period, the propofol was discontinued. If there was a need to start sedatives three times, the patient was sedated in the same manner as the daily awake group	Morphine and propofol to maintain Ramsay 3 - 4, assessed every 2 - 3 hours. Sedation was stopped and awakening assessed daily. In this regard, the patient had to be able to complete three of four tasks: open his eyes, follow with his eyes, shake hands, put out his tongue. After awakening, the sedative was reinitiated at half dose to maintain Ramsay 3 - 4. After 48 hours, propofol was replaced with midazolam
Yiliaz et al.^(21)^	Fentanyl for pain control, with target of BPS ≤ 6 and midazolam for agitation control with a target of Ramsay 3 - 4. Additional sedatives (diazepam, propofol and dexmedetomidine) could be used if the Ramsay target was not reached	Sedation interruption was employed at any time (without further details)
Mehta et al.^(22)^	Adjustment of opioid infusion and sedatives for achieving the target, as in the 2008 study	Daily sedation interruption was employed. If the patient could follow three out of four commands, the infusion was kept off at the discretion of the doctor and nurse. If there was a need for sedation or agitation or discomfort, then the doses instituted were half of the previous doses
Nassar Junior e Park^(23)^	Maintain without sedation. Analgesia with fentanyl. If the patient was agitated (SAS ≥ 5), the team searched for the causes of agitation, and delirium was treated with haloperidol. If the patient remained agitated, sedation was initiated with midazolam or propofol	Daily sedation interruption was employed until the patient could follow commands (open your eyes, follow with your eyes, shake hands and open your mouth). Sedatives and opioids were reinitiated at half the dose if agitated (SAS ≥ 5)

CrCl - creatinine clearance; SAS - Sedation Agitation Scale; RASS - Richmond Agitation Sedation Scale; RR - respiratory rate; SaO_2_ - arterial oxygen saturation; HR - heart rate; SBP: systolic blood pressure; ICP - intracranial pressure; BPS - Behavioral Pain Scale.

### Quality assessment

Generally, all studies had a low risk of bias, except for the blinding of participants and professionals, which was absent in all studies. The risk of bias was considered "uncertain" for the blinding of outcome assessors, as they could know into which sedation strategy patients were randomized. This risk was considered uncertain because the analyzed outcomes were goals (e.g., mortality) or because this assessment was not described in the study. Allocation concealment was not adequately described in one study;^(19)^ thus, the risk of bias was considered "uncertain" (Table 3).

**Table 3:** Risk of bias assessment

Study	Generation of random sequence	Concealment of allocation	Blinding of participants and professionals	Blinding of outcome assessors	Incomplete outcomes	Selective outcome reporting	Other sources of bias
Mehta et al.^(17)^	Low	Low	High	Uncertain	Low	Low	Low
de Wit et al.^(18)^	Low	Low	High	Uncertain	Low	Low	Low
Anifantaki et al.^(19)^	Low	Uncertain	High	Uncertain	Low	Low	Low
Strom et al.^(20)^	Low	Low	High	Uncertain	Low	Low	Low
Yiliaz et al.^(21)^	Low	Low	High	Uncertain	Low	Low	Low
Mehta et al.^(22)^	Low	Low	High	Uncertain	Low	Low	Low
Nassar Junior and Park^(23)^	Low	Low	High	Uncertain	Low	Low	Low

### Outcomes

**Figure 2:**
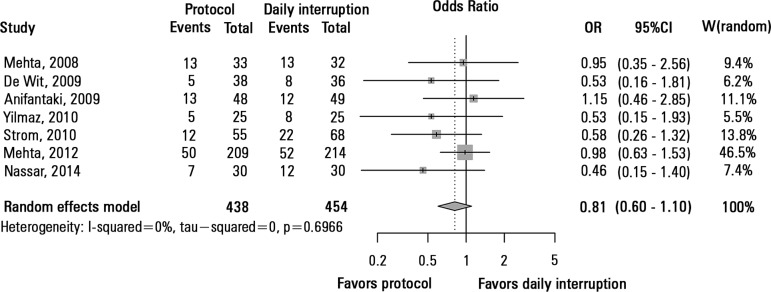
Mortality in the intensive care unit. OR - odds ratio; 95%CI: 95% confidence interval; W - weight of study.

ICU mortality was assessed in seven studies with a total of 892 patients. There were no differences in ICU mortality between the sedation protocol and daily sedation interruption groups (OR = 0.81, 95% CI 0.60 - 1.10; I^2^ = 0%) (Figure 2). Hospital mortality was assessed in six studies, with a total of 832 patients. There were no differences in hospital mortality between the sedation protocol and daily sedation interruption groups (OR = 0.89, 95% CI 0.66 - 1.19; I^2^ = 0%) (Figure 1S - http://www.rbti.org.br/content/imagebank/pdf/0103-507X-rbti-28-04-0444-suppl01-en.pdf).

**Figure 3:**
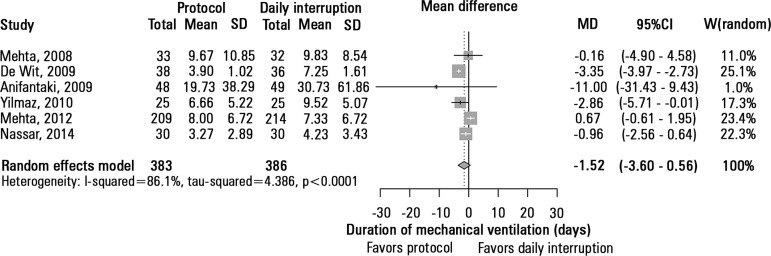
Time on mechanical ventilation. SD - standard deviation; MD - mean difference; 95%CI - 95% confidence interval; W - weight of study.

The duration of mechanical ventilation was assessed in 6 studies, which included 769 patients. The sedation protocols were not associated with any reduction in the duration of mechanical ventilation when compared to daily sedation interruption (MD = -1.52 days, 95%CI -3.60 - 0.56 days; I^2^ = 86.1%) (Figure 3). Three studies, totaling 266 patients, analyzed the free days of mechanical ventilation in 28 days. The sedation protocols were associated with an increase free days of mechanical ventilation, but this result was marked by significant heterogeneity (MD = 6.70 days; 95%CI 1.09 - 12.31 days; I^2^ = 87.2%) (Figure 2S - http://www.rbti.org.br/content/imagebank/pdf/0103-507X-rbti-28-04-0444-suppl01-en.pdf).

Intensive care unit length of stay was assessed in six studies, including 769 patients. Hospital length of stay was also assessed in six studies and included 851 patients. There were no differences in the duration of ICU length of stay between the sedation protocols and daily sedation interruption (MD = -2.41 days, 95%CI -6.37 - 1.54 days; I^2^ = 89.9%) (Figure 3S - http://www.rbti.org.br/content/imagebank/pdf/0103-507X-rbti-28-04-0444-suppl01-en.pdf). However, the sedation protocols were associated with a shorter duration of hospital length of stay (MD = -5.05 days, 95%CI -9.98 - -0.11 days, I^2^ = 69%) (Figure 4S - http://www.rbti.org.br/content/imagebank/pdf/0103-507X-rbti-28-04-0444-suppl01-en.pdf).

Accidental extubation and extubation failure were assessed in four studies, involving 661 patients. The sedation protocols were not associated with higher rates of accidental extubation (OR = 1.02, 95%CI 0.55 to 1.90; I^2^ = 0%) (Figure 5S- http://www.rbti.org.br/content/imagebank/pdf/0103-507X-rbti-28-04-0444-suppl01-en.pdf) or extubation failure (OR = 0.64, 95%CI 0.36 - 1.14; I^2^ = 0%) (Figure 6S-. http://www.rbti.org.br/content/imagebank/pdf/0103-507X-rbti-28-04-0444-suppl01-en.pdf) compared to daily sedation interruption. The occurrence of delirium was assessed in only three studies, for a total of 596 patients. Delirium was not more common in patients allocated to sedation protocols than in those allocated to daily sedation interruption (OR = 1.45, 95%CI 0.77 - 2.73; I^2^ = 42.6%) (Figure 7S - http://www.rbti.org.br/content/imagebank/pdf/0103-507X-rbti-28-04-0444-suppl01-en.pdf).

## DISCUSSION

This systematic review and meta-analysis suggests that there are no differences between sedation protocols that target light sedation levels and daily sedation interruption strategies regarding mortality, duration of mechanical ventilation and length of ICU stay. With the use of sedation protocols targeting lighter levels of sedation, the number of free days of mechanical ventilation was higher and the hospital stay was shorter. However, these findings were based on a small number of studies, in the case of time free of mechanical ventilation, and were marked by high heterogeneity within the two results.

The minimization of sedation is imperative when considering the deleterious effects of excessive sedation.^(4,5)^ Sedation protocols^(25)^ and daily sedation interruption^(8)^ have been studied for over 15 years and have shown significant benefits in terms of outcomes^(10,11)^ and safety with respect to adverse events, such as accidental extubation, extubation failure^(9,25)^ and long-term psychological outcomes.^(12)^ The results of our meta-analysis do not suggest significant differences between the two approaches in regard to important outcomes. In addition, our meta-analysis suggests low occurrences of accidental extubation and extubation failure, which are two common fears when addressing sedation reduction strategies.^(26,27)^

Another meta-analysis that aimed to assess the effectiveness of daily sedation interruption conducted a sub-analysis that compared it with the use of sedation protocols with regard to the duration of mechanical ventilation. The comparison also revealed no differences between the two approaches regarding this outcome.^(28)^ Our meta-analysis included two studies that were not included by this other review. Anifantaki et al.'s study^(19)^ was excluded because the authors considered that the protocol group represented "usual care" of the unit where the study was conducted. Unlike the Cochrane review, we decided to include the first study because the protocol effectively describes targeting a light sedation level.^(19)^ In our opinion, "usual care" refers to the decision of the unit to infuse sedatives according to physician's orders and without set targets, as effectively occurred in the other studies included^(8,9,29)^ and in the other meta-analysis.^(28)^ Strom et al.'s study^(20)^ was excluded because the authors contemplated interruption of sedation in both groups. However, we decided to include this study because daily sedation interruption became part of the protocol only for patients who were still uncomfortable after administration of haloperidol and four attempts to suspend propofol infusion, used to control agitation.^(20)^ Therefore, we considered that sedation interruption was part of the protocol and not the protocol itself, as occurred in the other group.

Despite the apparent equivalence of daily sedation interruption and the protocols regarding outcomes and safety, three additional factors should be addressed. The first and foremost factor refers to nursing workload. While one of the studies included in this meta-analysis suggested the need for one professional more per patient to meet the demands of the same in the sedation protocol group,^(20)^ a Brazilian study showed no differences in nursing workload between groups during the first five days of mechanical ventilation.^(23)^ The second factor relates to the expectations and preferences of the professionals responsible for patient care. While approval of the sedation protocol was similar for physicians and nurses who participated in another study included in the review,^(22)^ nurses' approval of daily sedation interruption was much lower than that of doctors. Nurses considered that the sedation protocol was easier to use and allowed greater patient comfort.^(27)^ The third factor relates to the experience and knowledge of a strategy, which, as would be expected, is associated with a greater likelihood of employing that strategy.^(30)^

The results of this meta-analysis should be interpreted with caution. Despite an extensive database search, only seven studies met the previously established inclusion criteria. Six of the seven studies were single-center and included few patients. Therefore, their results may not be valid in centers with different profiles. The variation in study characteristics is evident from the high statistical heterogeneity found in the analysis of outcomes involving time (i.e., duration of mechanical ventilation, ICU stay and hospital stay). Despite having a common target of sedation, both the protocols and the daily interruption strategy were performed differently from one study to another (Table 2), which could lead to discrepancies in results. We believe that data from ongoing studies (NCT01728558, NCT02040649) should shed more light on our findings in the coming years.

## CONCLUSION

Sedation protocols and daily sedation interruption appear to be equivalent as strategies targeting lighter sedation levels, although it should be noted that the target of sedation should be the primary goal of management in most patients under mechanical ventilation.
