# Supplementary material for: Sedation protocols versus daily sedation interruption: a systematic review and meta-analysis
Source: Rev Bras Ter Intensiva. 2016 Oct-Dec;28(4):444–51. doi: 10.5935/0103-507X.20160078 (PMC5225920; doi:10.5935/0103-507X.20160078)
Supplement: Supplementary file 1 [file rbti-28-04-0444-suppl01.pdf]

Sedation protocols *versus* daily sedation interruption: a systematic review and meta-analysis

Protocolos de sedação versus interrupção diária de sedação: uma revisão sistemática e metanálise

Antonio Paulo Nassar Junior, Marcelo Park

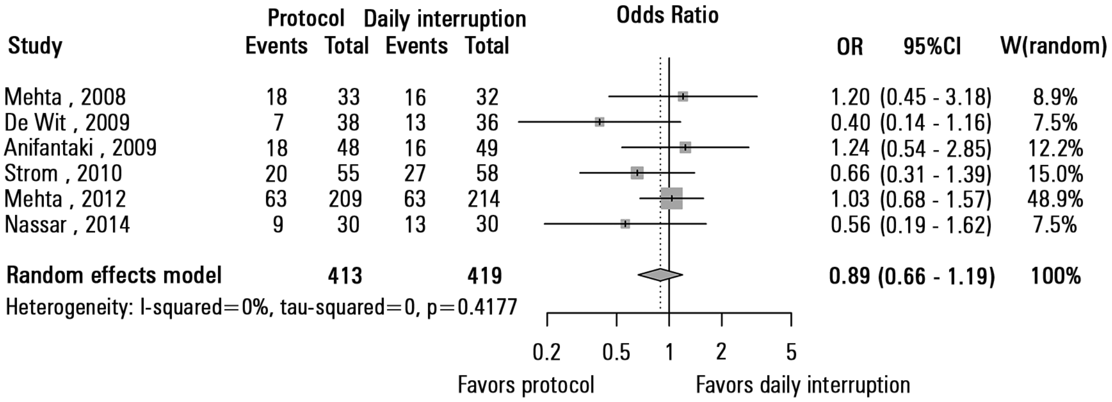

Figure S1 - Hospital mortality. OR - odds ratio; 95%CI - 95% confidence interval; W - weight of study.

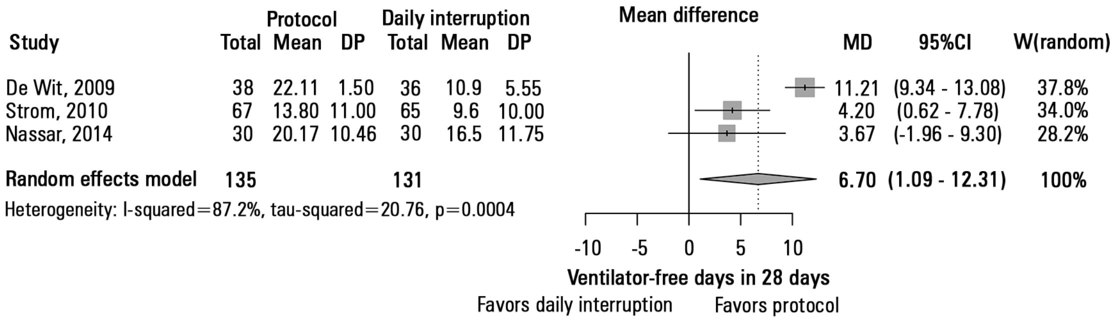

Figure S2 - Days free of mechanical ventilation in 28 days. SD - standard deviation; MD - mean difference; 95%CI - 95% confidence interval; W - weight of study.

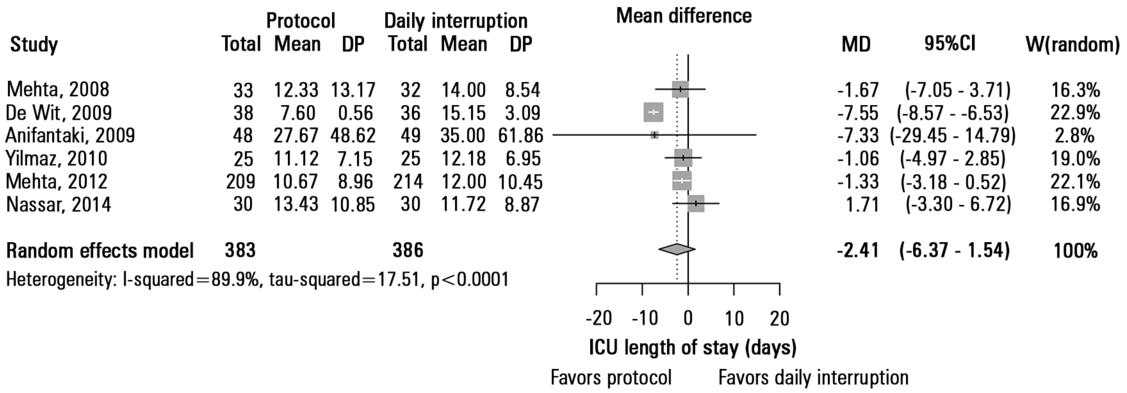

Figure S3 - Length of stay in the intensive care unit. SD - standard deviation; MD - mean difference; 95%CI - 95% confidence interval; W - weight of study; ICU: intensive care unit.

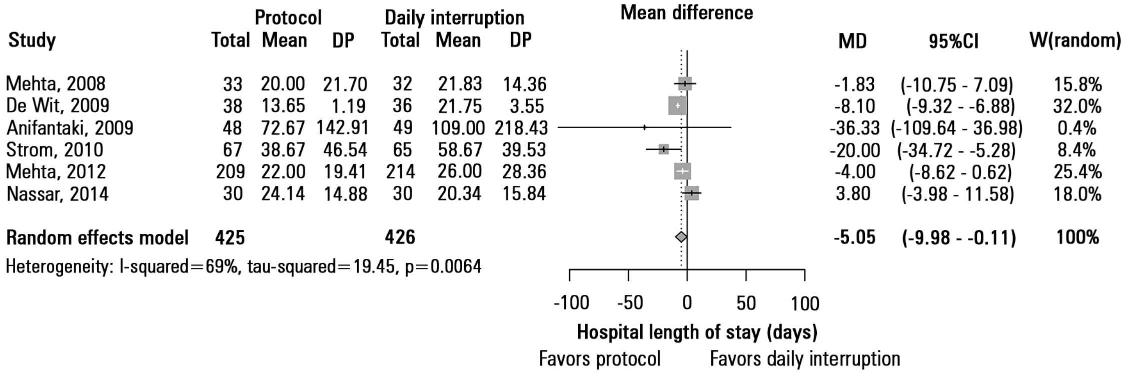

Figure S4 - Length of hospital stay. SD - standard deviation; MD - mean difference; 95%CI - 95% confidence interval; W - weight of study.

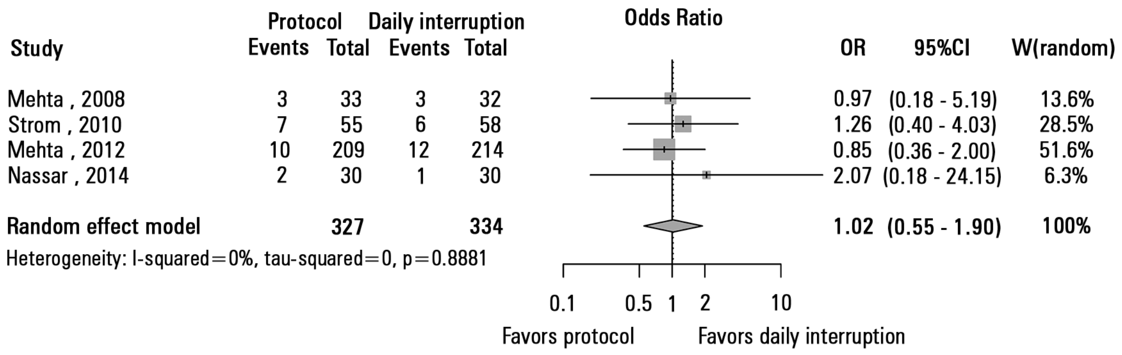

Figure S5 - Accidental extubation. OR - odds ratio; 95%CI - 95% confidence interval; W - weight of study.

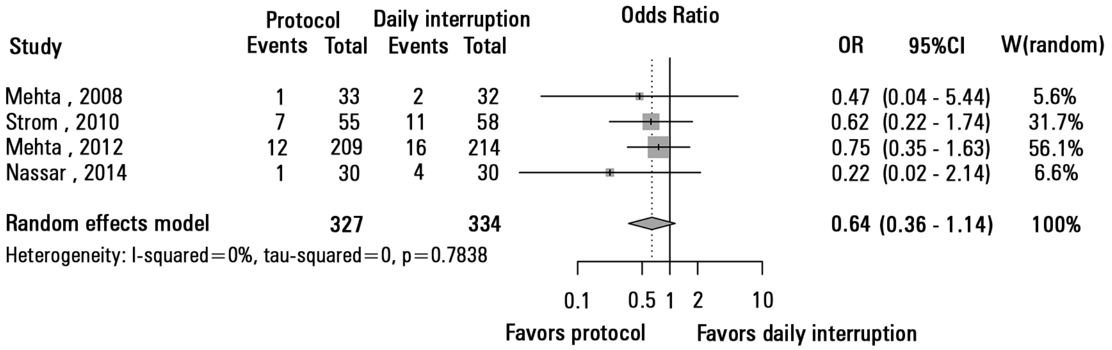

Figure S6 - Reintubation within 48 hours. OR - odds ratio; 95%CI - 95% confidence interval; W - weight of study.

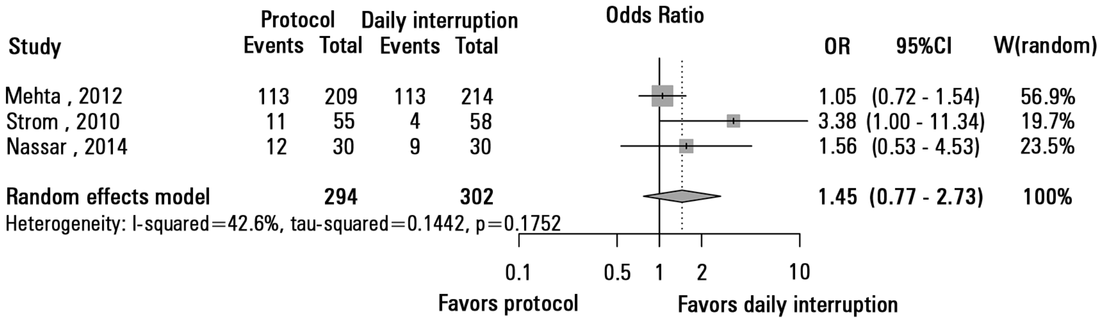

Figure S7 - Delirium. OR - odds ratio; 95%CI - 95% confidence interval; W - weight of study.
